# Supplementary material for: A cross-study transcriptional patient map of heart failure defines conserved multicellular coordination in cardiac remodeling
Source: Nat Commun. 2025 Oct 31;16:9659. doi: 10.1038/s41467-025-62219-6 (PMC12579254; doi:10.1038/s41467-025-62219-6)
Supplement: Supplementary file 1 — Supplementary Information [file 41467_2025_62219_MOESM1_ESM.pdf]

A cross-study transcriptional patient map of heart failure defines conserved multicellular coordination in cardiac remodeling

Jan D. Lanzer[1,4†], Ricardo O. Ramirez Flores[1,2†], José Liñares Blanco[2], Marco Steier[3,4], Ashraf Y. Rangrez[3,4], Norbert Frey[3,4] and Julio Saez-Rodriguez[1,2*]

1. Supplementary Information

1.1 Supplementary notes

Supplementary Note 1: Update of the consensus bulk HF signature

We compared the updated consensus signature with the previous version by assessing overlap of top gene sets and their classifier performance. Although the updated ranking consensus signature reordered the top 500 genes (Jaccard index 0.49, Supplementary Figure 2D), the classification performance these genes did not change significantly and remained high (mean AUC 0.962 vs 0.968 [old version], t-test p=0.235, Supplementary Figure 2E). The enrichment scores of the updated consensus signature also did not change significantly compared to the previous version (Supplementary Figure 2F). However, the number of genes reported under the adjusted Fisher combined test p-value $<10^{-5}$ increased from 1,809 to 4,518 substantially, including a higher gene coverage (increased from 14,041 to 16,036) (Figure 2C). Thus, the inclusion of new studies increased the robustness of the conserved events of deregulation captured by the gene ranking, while suggesting that classification performance cannot be substantially improved by adding additional data sets.

Supplementary Note 2: Comparison of pseudobulk expression matrices of single-nucleus studies.

Core studies had a median of 17,523 non-zero genes measured in each cell-type pseudobulked data and were calculated from a median of 744 cells with significant but not substantial differences in both, number of cells and genes, across studies (ANOVA adj. p-value < 0.001, Supplementary Figure 3A-C), reflecting expected differences at the technical level.

Ambient molecules from droplet-based single-nucleus experiments can influence gene expression profiles and affect the accuracy of cell-type specific disease signatures [1]. For the same reason, we quantified the levels of gene expression contamination in each pseudobulk profile (Supplementary Figure 4A-D). We assumed that for a pseudobulk profile of a given cell-type, a simple measure of the levels of contamination in its gene expression could be approximated from the expression of unexpected genes relative to expected genes. Here we used marker genes of the cell-type from which the pseudobulk profile was created to define the set of expected genes, and the marker genes of the other six cell-types as unexpected ones. Then, we defined a contamination score for each pseudobulk profile as a ratio between the read counts of background and marker genes. CM profiles had the lowest contamination scores (mean = 0.124) of all cell-types (t-test adj. p-value < 0.05), and in

all studies the gene expression profiles of vSMCs contained more contamination reads than marker genes (one-sample t-test adj. p-value < 0.05, null hypothesis: contamination score mean = 1, Supplementary Figure 4A, C). In addition, we observed that CMs were the cells that contributed the highest fraction (mean fraction of 0.4, Supplementary Figure 4B) of contaminating reads to other cell-types (t-test adj. p-value < 0.05). Two studies provided background corrected gene expression data and we observed differences in the contamination scores in all cell-types between background corrected and not-corrected studies (t-test adj. p-value < 0.05). However, depending on the cell-type analyzed, background correction would increase (Myeloid, Fib, vSMCs) or decrease (CM, Endo, PC, Lymphoid) contamination (Supplementary Figure 4D). Our results point to a shared technical limitation in these studies that should be taken into account when comparing gene expression profiles between non-failing and failing hearts.

Supplementary Note 3: Multicellular factor analysis of individual studies

We observed on average that the multicellular programs of individual studies captured 17% of explained variance associated with HF (ANOVA adj. p-value <= 0.05, Supplementary Figure 5C), 20% with left ventricle ejection fraction and 5% to age (linear model adj. p-value <= 0.05, Supplementary Figure 5D). Other patient covariates such as sex or body mass index had no associations with the multicellular space. Visualization of samples in a 2-dimensional Uniform Manifold Approximation and Projection (UMAP) space built from the multicellular programs showed clear separations of failing and non-failing hearts in all studies (Figure 2G).

Supplementary Note 4: Experimental validation of fibroblast derived ligands in cardiomyocytes.

To validate our computational predictions of the gene regulatory effects of fibroblast’s ligands in CMs during HF, we selected three ligands expressed by Fibs (Bmp4, Mxra5, and Nrg1) and investigated their pro-hypertrophic effects in cell cultures of neonatal rat ventricular cardiomyocytes and the changes in gene expression they generated (Methods). Bmp4 and Nrg1 treatment upregulated the cardiomyocyte stress response markers *Nppa* and *Nppb* (Supplementary Figure 9A-B adj. p-value of t-test from linear mixed model < 0.05), supporting their involvement in hypertrophic signaling as predicted by our cell-cell communication analysis using gene expression data of MCP1. In contrast, Mxra5 upregulated *Nppa* but downregulated *Nppb* (adj. p-value of t-test from linear mixed model < 0.05), indicating a distinct regulatory mechanism than the one inferred in silico (Supplementary Figure 9A-B). Nrg1 did not affect fibrotic markers *Col1a1* or *Tgfb1*, whereas Bmp4 and Mxra5 selectively upregulated *Tgfb1* without altering *Col1a1* (adj. p-value of t-test from linear mixed model < 0.05), indicating potential pro-fibrotic roles (Supplementary Figure 9A-B). As expected, phenylephrine (PE), used as a positive control, robustly increased *Nppa*, *Nppb*, *Col1a1*, and *Tgfb1* expression, validating the experimental system (Supplementary Figure 9A–B, adj. p-value of t-test from linear mixed model < 0.05).

To further dissect ligand-specific signaling, we assessed the expression of predicted NicheNet targets within MCP1 following ligand treatment. For this analysis we selected genes with an MCP1 gene loading < -0.2 and a target weight >0 assigned by NicheNet, resulting in a list of 19, 22 and 16 target genes for Bmp4, Mxra5, and Nrg1, respectively. From these lists we selected ten, nine and nine genes for the respective ligands, based on a literature research to prioritize candidates with a possible functional relevance for hypertrophy. qPCR analysis revealed that Bmp4, Mxra5, and Nrg1 induced the expression of 6 out of 10, 2 out of 9, and 3 out of 9 of their respective selected predicted target genes, respectively (Supplementary Figure 9C, adj. p-value of t-test from linear mixed model < 0.05). From the 11 upregulated genes by Bmp4, Mxra5, and Nrg1, PE did not induce seven genes despite strongly inducing classical hypertrophy and fibrosis markers (Supplementary Figure 9C), suggesting that the identified ligand-target gene associations are often ligand specific. Among these specific interactions are the upregulation of *Nav2* and *Sacs* by Bmp4, with unknown function in cardiac fibrosis or hypertrophy, but could be functionally involved in cytoskeletal coherence (*Nav2*[2]) and mitochondrial function (*Sacs*). Nrg1-induced *Ifnar2* and *Lyr* could be linked to interferon response[3,4] and mitochondrial health[5] in cardiomyocytes, respectively, while Mxra5 might have protective effects on cell survival (*Mcl1*[6]) and anti-inflammatory effects (*Socs2*[7,8]). Together, these findings corroborate many ligand-target predictions and reveal canonical and non canonical ligand targets involved in cardiomyocyte hypertrophy and fibrosis. These results support the relevance of the selected ligands and their target genes in the multicellular program of HF and underscore the need for mechanistic studies in in vitro and in vivo models to elucidate their roles in cardiac pathology.

Supplementary Note 5: Enrichment of specialist, generalist, and acquired generalist functions of fibroblasts to MCP1 and bulk data

To characterize which expression group (specialist, generalist or acquired generalist) predominates in the Fib component of MCP1, we compared their respective gene loadings. The acquired generalist program was assigned the highest median loading (Wilcoxon's tests, vs. generalist genes $p=2.9e^{-}11$, Supplementary Figure 11G), suggesting that the key genes in the MCP1 were characterized by variability between states but accommodating a generalist expression pattern by upregulation across multiple states. Next, we enriched these programs in the HF consensus bulk signature and found that the acquired generalist program displayed the highest enrichment scores (Supplementary Figure 11H), suggesting that their importance generalizes to a larger HF cohort and can be detected in bulk signatures.

Supplementary Note 6: Evaluation of sets of cell-type marker genes for cell deconvolution from bulk transcriptomics

When deconvoluting diseased samples, we found that markers regulated at the compositional level performed better than every other marker set (root mean squared error [RMSE]: 0.117, paired wilcoxon-test p-value = 0.002; Correlation: 0.895, paired wilcoxon-test p-value = 0.002; Figure 5E). For the deconvolution of healthy samples, this effect was mitigated. When comparing deconvolution

errors between cell types, we found that the compositional genes also lowered the RMSE compared to molecular genes in all cell types except for lymphoid cells and pericytes (RMSE, paired wilcoxon-test p-value <0.05) (Supplementary Figure 12D). A downstream goal of bulk deconvolution is the assessment of compositional changes between conditions. We found that molecularly regulated markers failed to reliably predict compositional changes (global average F1-score of 0.44) while compositional genes performed best (global average F1-scores 0.64) (Supplementary Figure 12E). Taken together, we found that molecularly regulated cell type markers are poor indicators of cell type composition in disease, and thus identifying compositionally regulated cell type markers can improve deconvolution results.

Supplementary Note 7: Projection of supporting studies into the HF multicellular patient map

Other supporting studies let to insights regarding the MCP2, where we observed differences in activation of MCP2 across distinct physiological time-points upon myocardial infarction aligned to the trajectory of HF samples where fibrotic heart tissues located in between ischemic and control tissues (ANOVA, p-value = 0.00005; t-test, adj. p-value < 0.05; Supplementary Figure 13A), potentially related to the activation of apoptotic processes of this multicellular program. A less clear separation between congenital heart disease samples and donor hearts across MCP2 was observed (t-test, p-value = 0.07; Supplementary Figure 13B).

Finally, we projected data of two mice models of heart disease, Angiotensin II-Induced (AngII) and transverse aortic constriction (TAC), to evaluate to what extent the mice phenotypic characteristics of cardiac hypertrophy and failure were aligned to HF patients (Supplementary Figure 13C-D). We did not capture associations between the activation of MCP1 and MCP2, and the disease progression of the TAC model or AngII mice representing HF. These results showed a disagreement between the major axes of disease variability between mice and human samples.

Supplementary Note 8: User guide for ReHeat2 platform

ReHeat2 is an interactive web-based platform that enables the exploration of gene expression changes in HF through the integration of bulk and single-nucleus transcriptomics data. The platform, accessible at https://www.saezlab.shinyapps.io/reheat2/, allows users to access the built resource through two functional modules. The first module is a gene query that allows the examination of gene-specific expression patterns to investigate shifts in cell-type composition, and assess molecular reprogramming within individual cell types in response to HF. The second module is an interactive network that can be used to study a given cell-type pair of interest for their estimated communication events.

Users initiate their query in the first functional module by selecting genes of interest (Supplementary Figure 14A). The platform then provides a comprehensive overview of their expression patterns in HF,

integrating evidence from bulk and single-nucleus transcriptomic datasets (Supplementary Figure 14B). Bulk transcriptomic data are first displayed as study-specific log fold changes (HF vs. NF), illustrating the variability in the direction of regulation across independent studies. To assess the statistical significance of gene deregulation, the platform incorporates the consensus bulk HF signature, ranking genes based on their combined p-value across datasets. This allows users to evaluate the strength of association between their queried genes and HF across the large bulk cohort of patients. Beyond assessing differential expression in bulk tissue, ReHeat2 then provides insights into how transcriptional changes are coordinated at the cellular level. The next section examines whether the upregulation of a gene is primarily driven by shifts in cell-type composition or by intrinsic molecular deregulation. Cell-type-specific expression patterns are visualized to determine whether a gene is a marker of a particular cell type, which, in conjunction with compositional changes, can indicate regulation at the compositional level. To assess molecular regulation, the platform queries MCP1 to identify gene loadings per cell type, providing insight into the contribution of intrinsic transcriptional changes to overall gene expression shifts. Then, results are summarized in an automated text to aid with the interpretation of the plots. For example, a query of *MXRA5*, *POSTN*, and *NPPA* reveals that all three genes are significantly upregulated in bulk heart failure datasets. Single-nucleus data further resolve these changes at the cellular level, showing that *NPPA* is primarily upregulated within cardiomyocytes (molecular shift), while *MXRA5* and *POSTN* are upregulated in Fibs, reflecting a combination of compositional and molecular shifts. By integrating these complementary analyses, ReHeat2 enables a nuanced interpretation of transcriptional regulation in HF, facilitating hypothesis generation.

The second functional module enables querying the multicellular network where users can explore specific cell type pairs and their estimated communication events (Supplementary Figure 14C). The displayed network highlights the most significant edges, providing an overview of key interactions in failing or non-failing hearts. Selecting an edge of interest automatically generates a series of plots to estimate crucial communication events that may mediate the observed dependency in the network. The platform offers i) an overview of relevant ligand-receptor pairs, ii) ligand prioritization based on both expression scores and NicheNet regulatory potential, and iii) an analysis of the top downstream target genes potentially influenced by the ligand (Supplementary Figure 14D). By studying these interactions, researchers can gain insights into the estimated molecular mechanisms driving cell type interactions in HF.

Thus, the ReHeaT2 web platform enables a quick consultation of the combined evidence of the re-analyzed and combined 34 HF studies to facilitate hypothesis generation and translational insights.

1.2 Supplementary figures

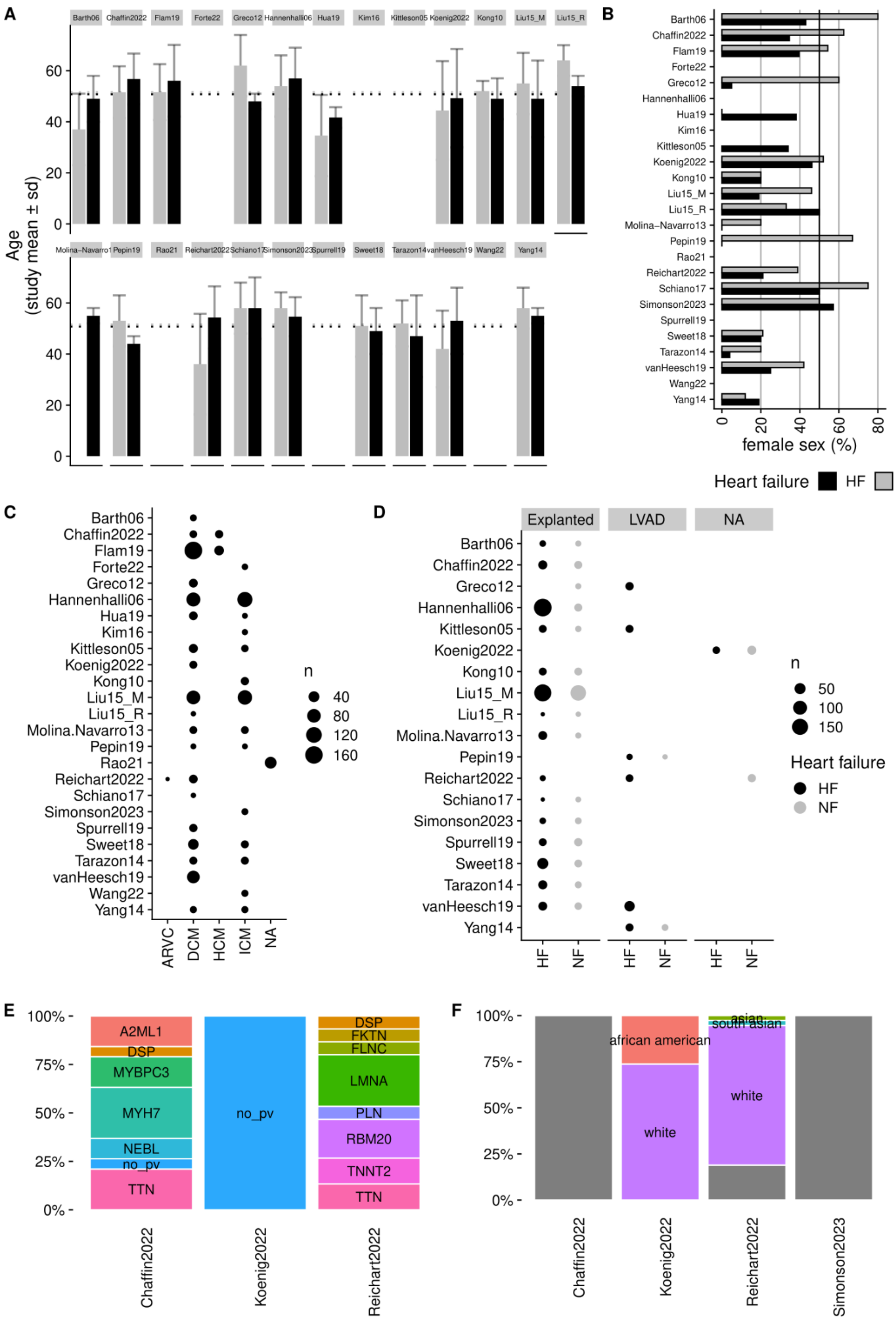

A
B
C
D
E
F
Age (study mean ± sd)
female sex (%)
Heart failure
HF
n
Explanted
LVAD
NA
Barth06
Chaffin2022
Flam19
Forte22
Greco12
Hannenhalli06
Hua19
Kim16
Kittleson05
Koenig2022
Kong10
Liu15_M
Liu15_R
Molina−Navarro13
Pepin19
Rao21
Reichart2022
Schiano17
Simonson2023
Spurrell19
Sweet18
Tarazon14
vanHeesch19
Wang22
Yang14
ARVC
DCM
HCM
ICM
NA
HF
NF
A2ML1
DSP
MYBPC3
MYH7
NEBL
no_pv
TTN
FKTN
FLNC
LMNA
PLN
RBM20
TNNT2
african american
white
asian
south asian

Supplementary Figure 1. Extended metadata presentation.

A-D) Presenting metadata per HF core study, including A) age distribution, B) sex distribution, C) HF etiology, D) Reason for biopsy. Bars in A represent means with error bars indicating standard deviation.

E) Comparing the pathogenic variants of HF patients with familial or genetic cardiomyopathy per single-nucleus study. PV = pathologic variant.

F) Comparing reported race of patients per single-nucleus study.

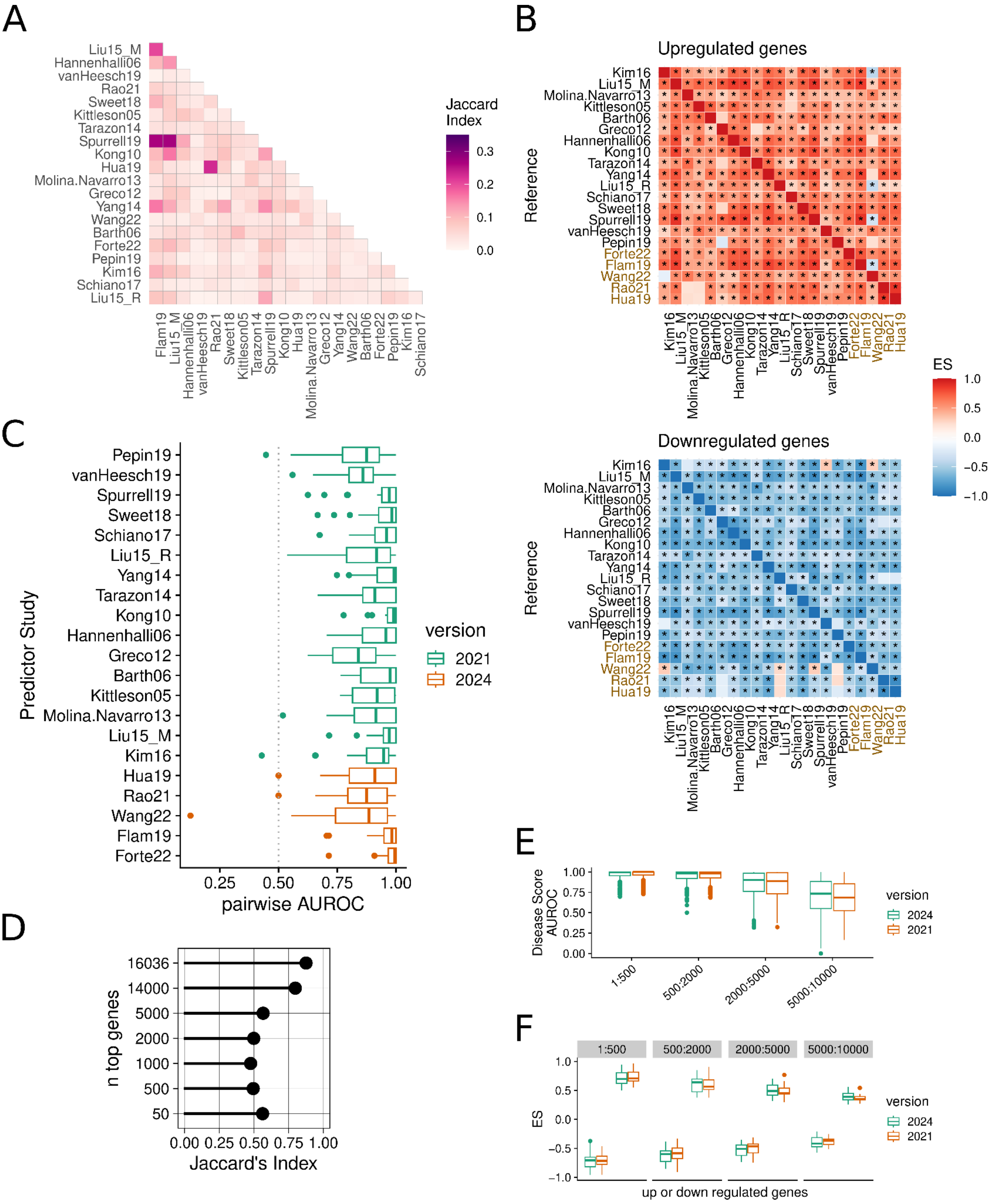

Supplementary Figure 2. Expansion of the consensus bulk transcriptional signature of HF

A. Pairwise quantification of the overlap of the top 500 differentially expressed genes between failing and non-failing hearts between core HF bulk studies using the Jaccard index.

B. Enrichment score (ES) of the top 500 differentially expressed genes between failing and non-failing hearts of each study in the sorted gene-level statistics list of each other study. Colored study names are

new studies added. Stars indicate an adjusted p-value < .05.

C. Comparison of the updated consensus ranking with the previous version. Jaccard indices were calculated between different number of top genes.

D. Comparison of classification via disease scores calculated with gene sets of different size from the updated and previous ranking.

E. Comparison of enrichment scores of top genes extracted from the consensus rankings in individual studies.

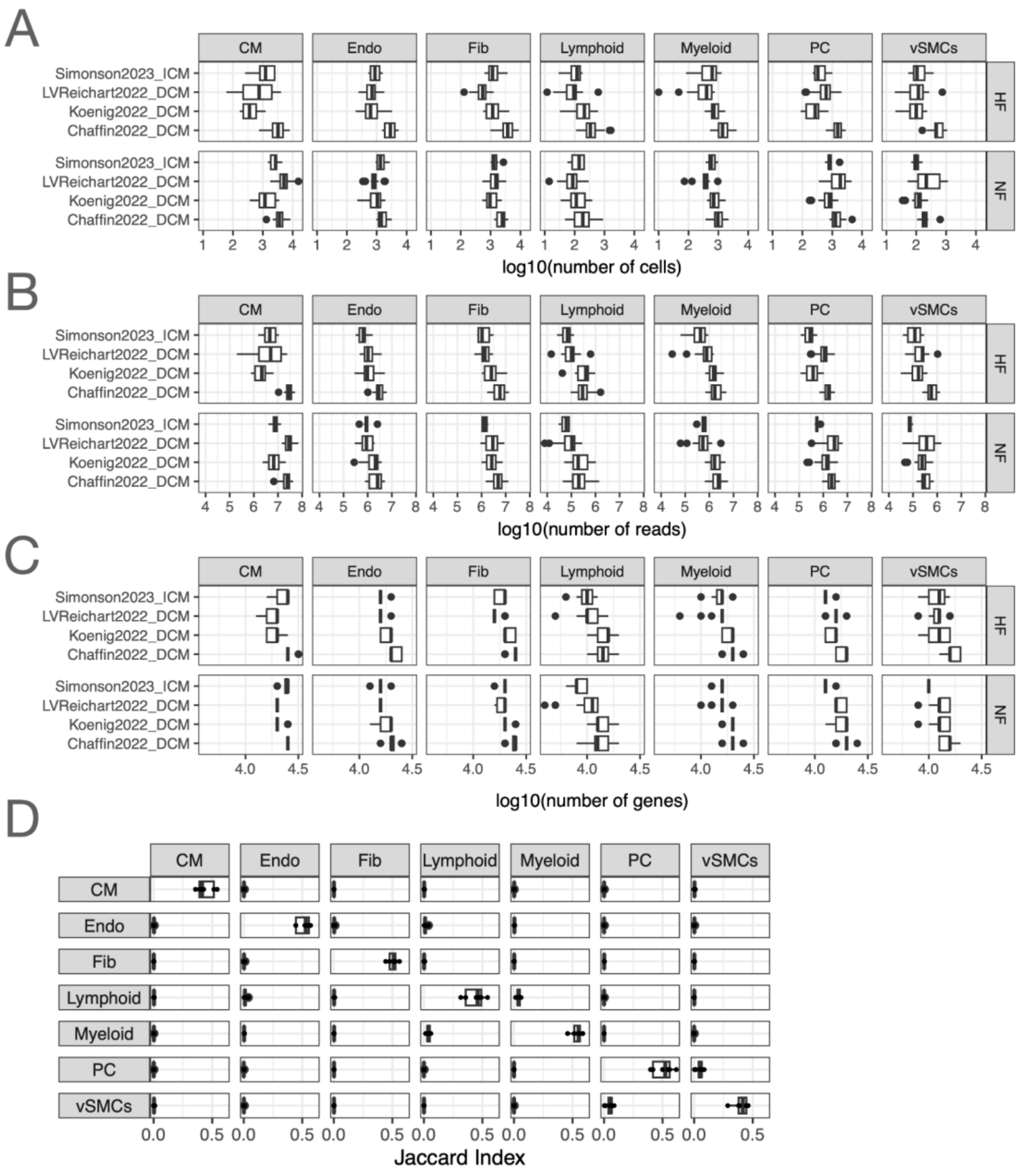

Supplementary Figure 3. Quality of pseudobulk expression profiles of the collection of core single-nucleus (SN) transcriptomics studies.

A-C. Distribution of A) the log10 (number of cells) used to calculate the pseudobulk expression profile, B) the number of reads, and C) the number of genes

D. Distribution of Jaccard Indices representing the pairwise similarities between sets of gene expression markers of cell-types (Methods). Each dot represents a comparison between the markers of cell-type X with markers of cell-type Y for every combination of SN-core studies.

In all panels Cardiomyocytes (CM), fibroblasts (Fib), pericytes, and endothelial (Endo), vascular smooth muscle (vSMCs) cells.

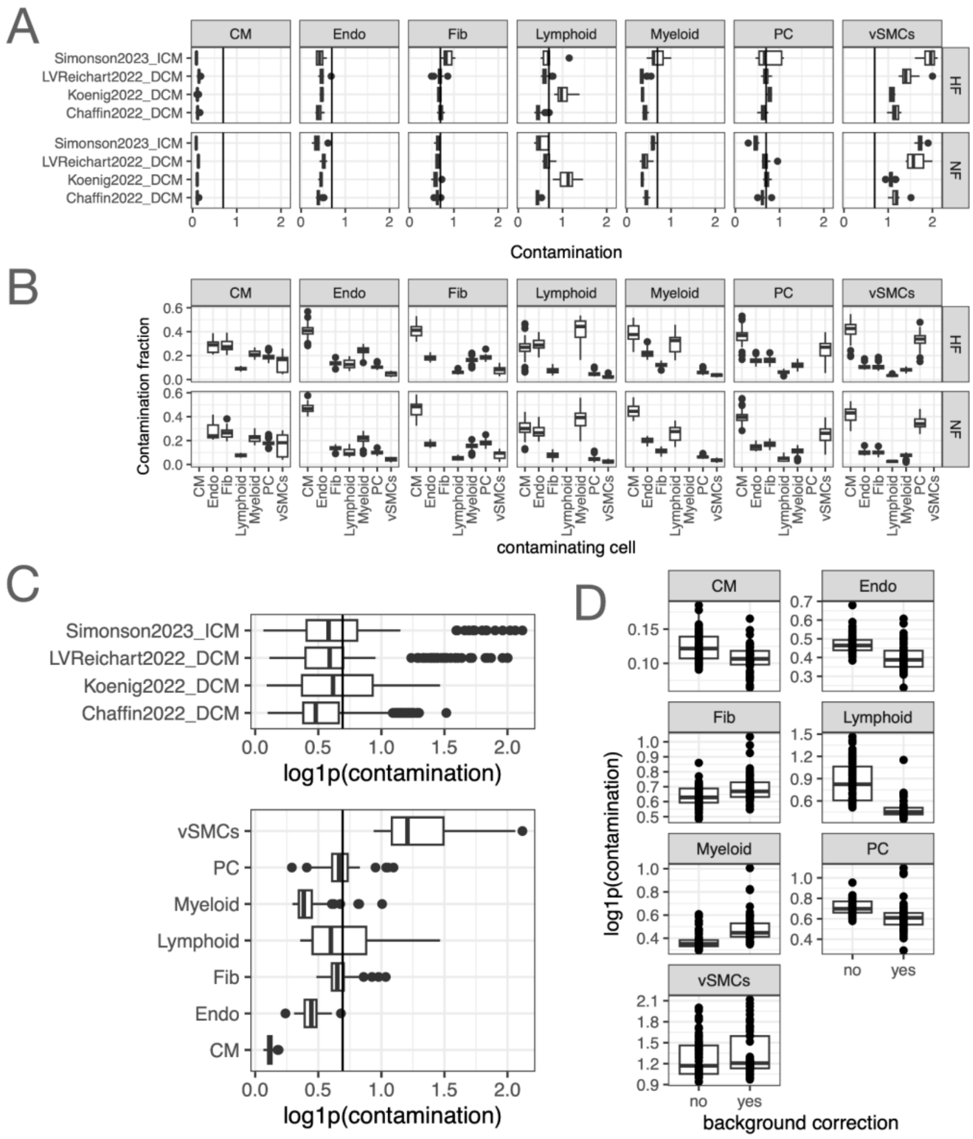

Supplementary Figure 4. Contamination of pseudobulk expression profiles of the collection of core single-nucleus (SN) transcriptomics studies.

A. Distribution of the contamination score of the tissue samples of each core SN study, divided by cell-type as defined by our ontology and disease status. The line denotes the score where the amount of cell-type reads and contaminating reads is identical.

B. Read contamination fraction of each cell-type in the x-axis to the expression profile of the cell-type in the header, across studies.

C. Distribution of the contamination scores of each tissue sample across SN-core studies (upper) or cell-types (lower). The line denotes the score where the amount of cell-type reads and contaminating reads is identical.

D. Distribution of the contamination scores of each tissue sample across cell-types separated in two groups based on previous background correction of the study.

In all panels Cardiomyocytes (CM), fibroblasts (Fib), pericytes, and endothelial (Endo), vascular smooth muscle (vSMCs) cells.

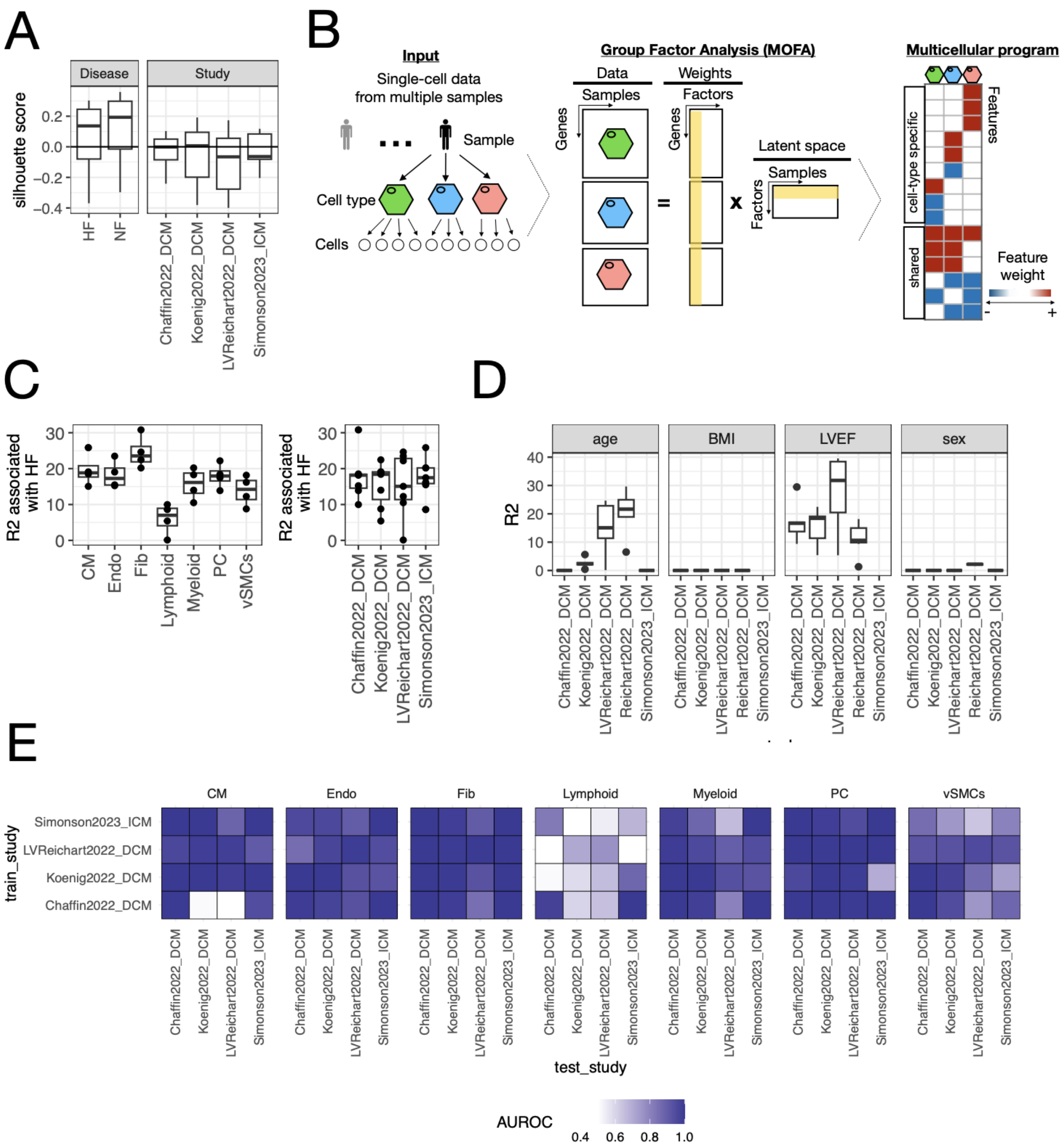

Supplementary Figure 5. Comparison of the single-nucleus (SN) core studies from compositions and multicellular programs

A. Distribution of silhouette scores of patient heart samples of SN core studies grouped by disease status (left) and study (right).

B. Multicellular factor analysis leverages single-cell data from multiple samples to infer multicellular programs (MCPs). First, pseudobulk expression matrices are generated for each cell type, creating a multi-view representation of the data across samples, where each view reflects the aggregated gene expression profile of a specific cell type. Next, group factor analysis, as implemented in MOFA, is applied to infer a latent space that captures sample variability while integrating information across all cell types simultaneously. Each latent component represents an MCP, describing coordinated gene expression changes across cell types, which can reflect shared or cell-type-specific processes.

C. Percentage of explained variance (R2) associated with HF (HF) captured by the multicellular factor analysis models fitted to each study independently. Each dot represents one study (left) or one cell-type (right).

D. Percentage of explained variance (R2) associated with age, body mass index (BMI), left ventricular ejection fraction (LVEF) and sex captured by the multicellular factor analysis models fitted to each study independently.

E. Area under the receiver operating characteristic curve (AUROC) of pairwise predictions of disease classifiers built from all core SN-studies using cell-type specific information within multicellular

programs.

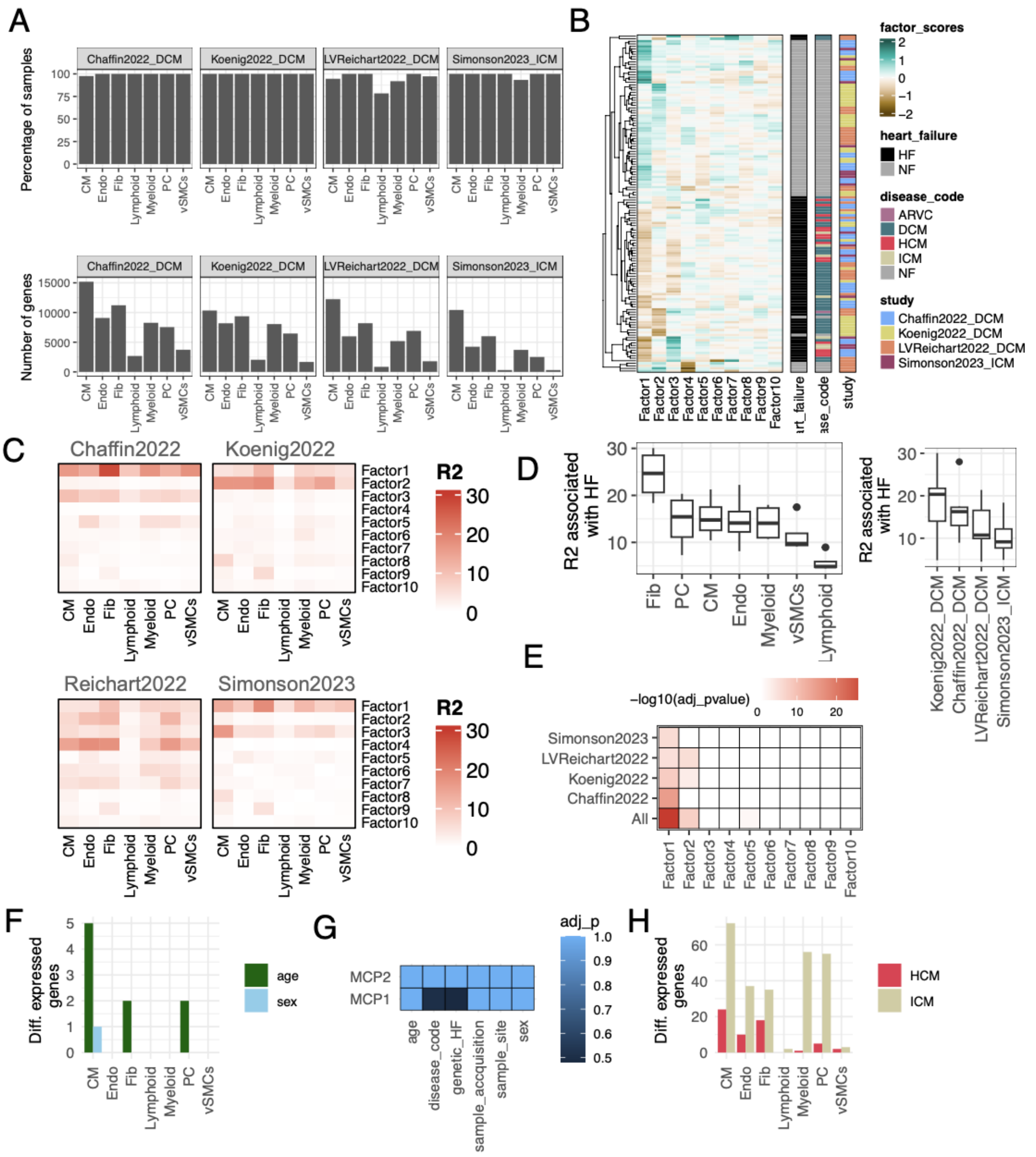

Supplementary Figure 6. Quality metrics of joint multicellular factor analysis of single-nucleus (SN) core studies

A. Percentage of patient samples and number of genes included in the multicellular factor analysis model separated by study

B. Hierarchical clustering of the patient-level values of the 10 multicellular programs inferred in the joint multicellular factor analysis model. Annotations of disease status, etiology, and study of origin are provided.

C. Percentage of explained variance (R2) that each multicellular program captured for each cell-type across studies.

D. Percentage of explained variance (R2) associated with HF captured by the joint multicellular factor analysis model. Each dot represents one study (left) or one cell-type (right).

E. -log10 of the adjusted p-values of an analysis of variance used to test for association between the disease status of patients and their multicellular program activation across all studies and with their union.

F. Number of genes whose variability in expression can be associated significantly with an interaction between HF and age or sex. Linear mixed models, adjusted p-value < 0.05.

G. Adjusted p-values of associations, using linear mixed models, between MCP1 and MCP2 scores in HF samples with reported clinical variables.

H. Number of genes whose variability in expression in HF patients can be associated significantly with a difference in etiologies. Linear mixed models, adjusted p-value < 0.05.

In all panels Cardiomyocytes (CM), fibroblasts (Fib), pericytes, and endothelial (Endo), vascular smooth muscle (vSMCs) cells. Heart failure (HF), and non-failing (NF) hearts.

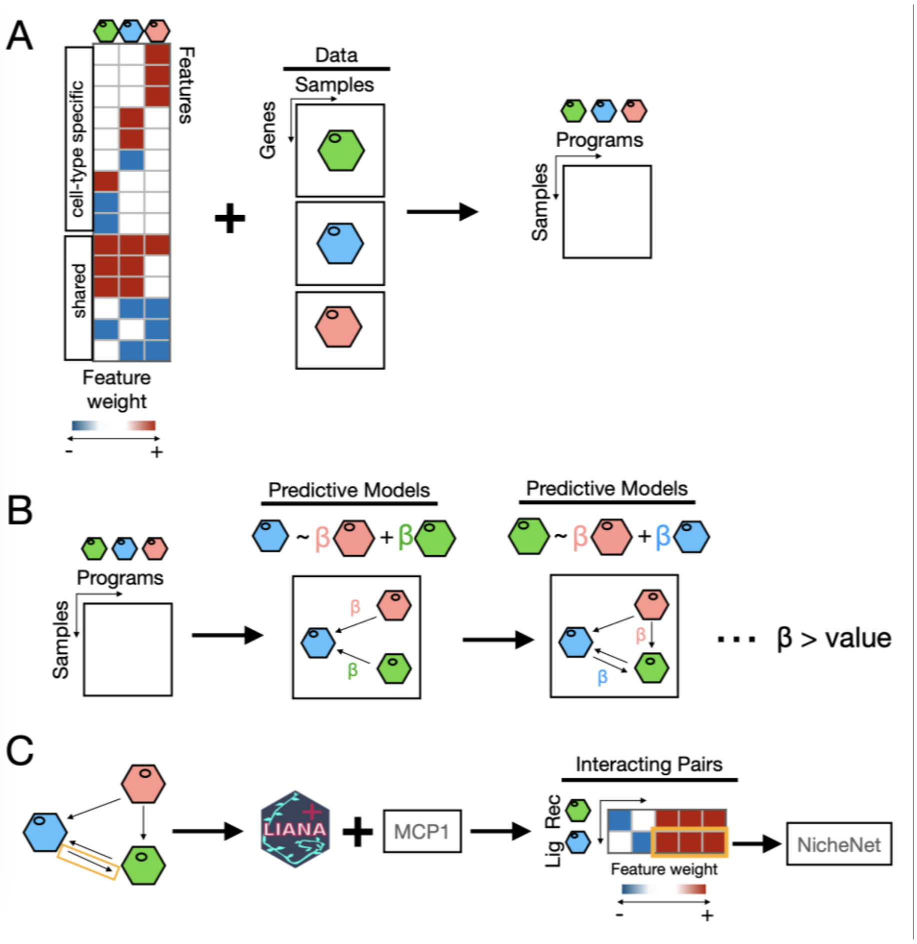

Supplementary Figure 7. Framework to infer the blueprint of coordination from multicellular programs and potential cell-cell communication.

A. Given a multicellular program, the activation of cell-type specific components can be inferred using enrichment methods.

B. The co-expression of the cell-type specific components of a multicellular program can then be represented as a network, here referred to as multicellular coordination network. In this network, each edge represents to what extent the expression profile of a given cell-type can predict the gene expression of the other. To build the network we fitted linear models where the target variable was the enrichment score of one cell-type and the predictors were the enrichment scores of all other cell-types. We used the model fit (R^2) and the coefficient estimated for each predictor to estimate the weight or importance of each edge. We kept all edges with weights larger or equal to 0.2.

C. For selected important edges (i.e. with high weights), we used LIANA+ to select from the multicellular program represented in the network, all potential interacting pairs of ligand and receptors, taking into account the edge directionality and feature weight (i.e. ligands and receptors from the predictor and predicted cell-type, respectively). Ligand-receptor pairs with coherent signs of feature weights (i.e. the feature weights of both the ligand and receptor were all positive or negative) then are selected as relevant interactions. Ligands from these interactions and the expression profile of the predicted cell-type are then used as input for NicheNet.

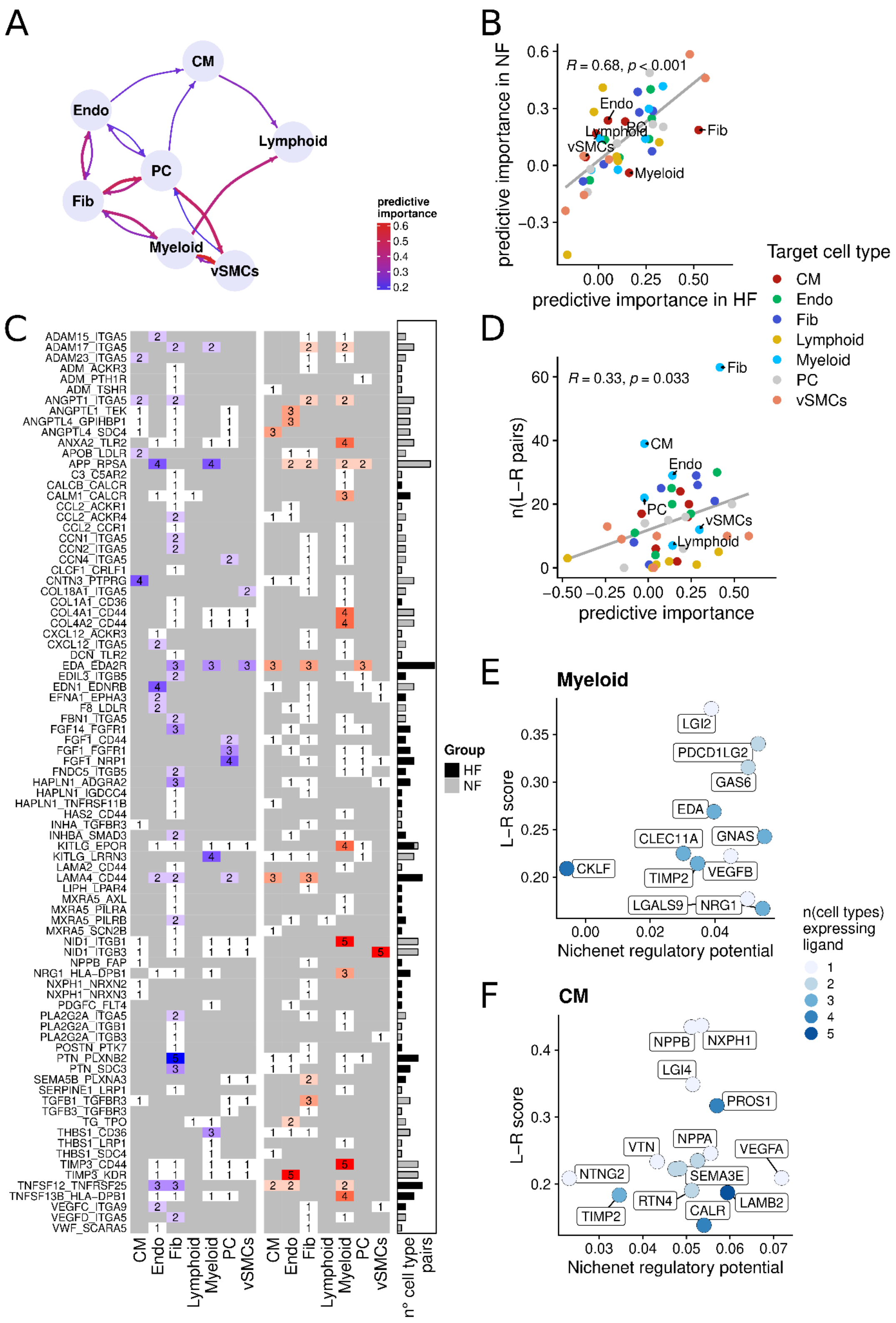

Supplementary Figure 8. Extended cell communication results

A. Multicellular coordination network of non-failing (NF) processes captured by MCP1, where each arrow describes how important the expression of a given cell-type is to predict the expression profile of another one (Methods). Predictive importances come from linear mixed models of cell-type signatures of MCP1. Importances below 0.2 were not included.

B. Comparing the predictive importances of the MCP1 expression in patients with heart failure (x-axis) and controls (y-axis). Each dot represents a directed cell type pair with target cell types colored. R, Pearson's correlation coefficient.

C. Heatmap showing top 5% of multicellular ligand-receptor interactions in MCP1. Numbers indicate the

number of cell type pairs. Left and right heatmaps display cell type pair counts of ligands and receptors, respectively. Bar graph displays number cell type pairs colored by disease group.

D. Comparing the predictive importance of MCP1 expression in non-failing patients (x-axis) with the number of ligand receptor pairs inferred. Each dot represents a directed cell type pair with target cell types colored. R, pearson's correlation coefficient.

E. Nichenet results for top L-R pairs between Myeloid and Fibroblasts. NicheNet derived a regulatory potential (corrected AUPR, x-axis) of a given ligand to deregulate a gene signature which here represents extreme gene loadings of the target cell type taken from the MCP1. L-R score (y-axis) represent the mean gene loadings of the ligand and the receptor of a given cell type pair. If a ligand connected with multiple receptors, the median L-R score was calculated. Color represent the number of cell types that express this ligand in HF.

F. Nichenet results for top L-R pairs between CM and fibroblasts.

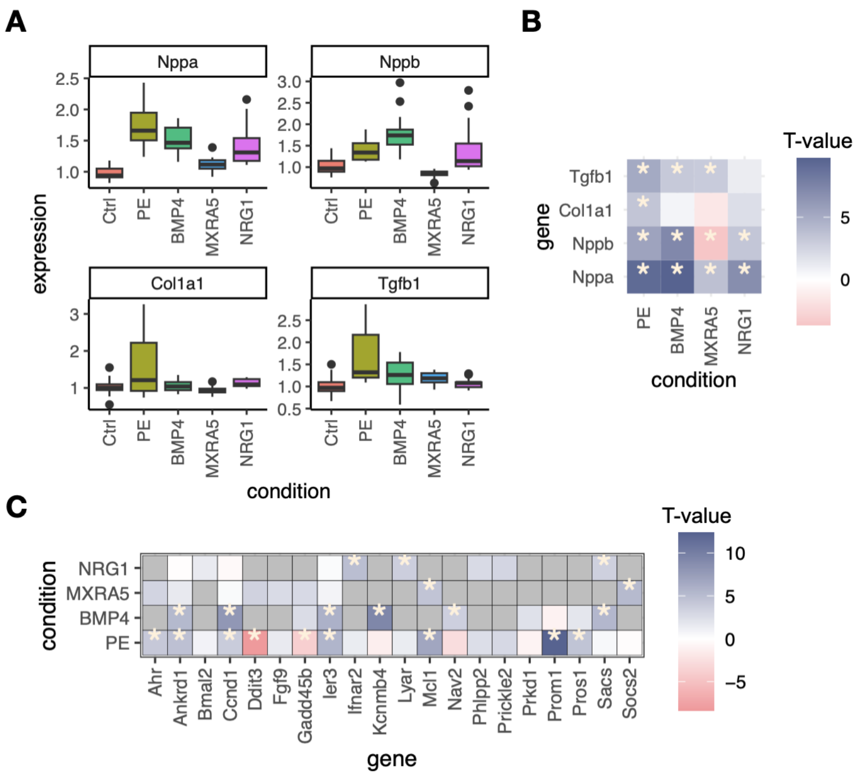

Supplementary Figure 9. Experimental Validation of fibroblast ligands inducing stress and hypertrophy in cardiomyocytes

A. Neonatal rat ventricular cardiomyocytes (NRVCMs) were treated for 24 h with Bone Morphogenetic Protein 4 (BMP4), Matrix Remodeling Associated 5 (MXRA5), or Neuregulin 1 (NRG1), and analyzed their effect on gene expression by quantitative real-time PCR (qPCR) for markers of hypertrophy (Nppa, Nppb) and fibrosis (Col1a1, Tgfb1). Phenylephrine (PE) treatment was used as a positive control. No treatment was used as a negative control (Ctrl).

B. Differential expression statistical testing between each NRVCM treated condition and control experiments. Markers of hypertrophy and fibrosis are highlighted

C. Differential expression statistical testing between each NRVCM treated condition and control experiments. Downstream signaling target genes as predicted by NicheNet are presented.

In all panels, data represent 18 measurements performed in three independent experiments, each with six technical replicates. In B and C, t-values extracted from the t-test of the coefficient of the fixed effect term (condition) from a linear mixed model of each target gene expression, with a random effect of experiments. Stars denote adjusted p-values (BH procedure) below or equal to 0.05. Grey tiles denote not performed tests.

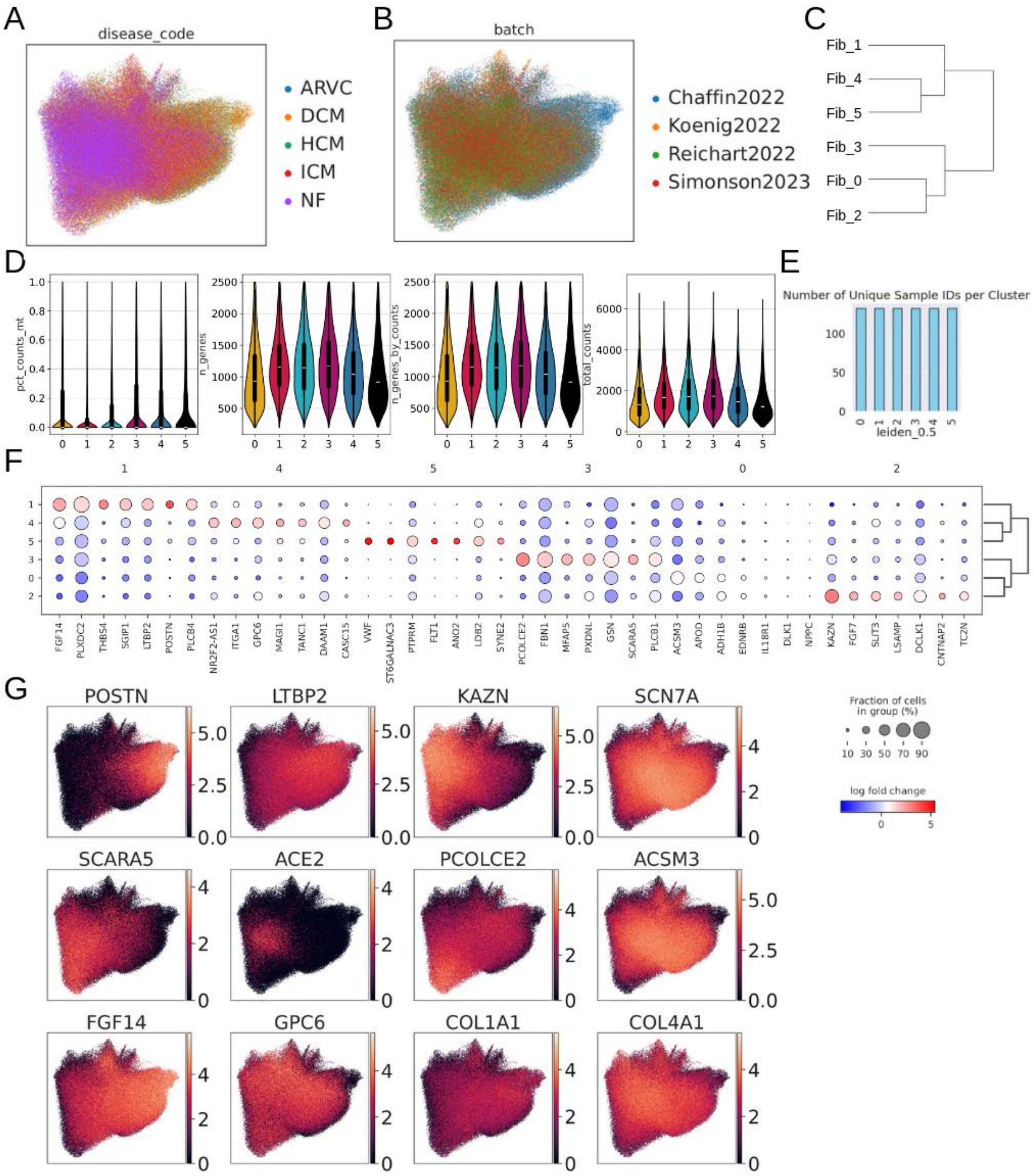

Supplementary Figure 10. Integrating fibroblasts single-nucleus RNAseq data.
A+B) UMAP embedding colored by disease code (A) and batch (B).
C) Dendogram of hierarchical clustering of fibroblast states.
D) Panels showing mitochondrial genes as percentage for each cell, number of unique genes per cell, number of unique genes with at least 10 counts per cell, and total counts per cell.
E) Number of unique patients per cluster.
F) Dot plot representing state marker expression per cell state.
G) UMAP visualization of expression of selected marker genes.

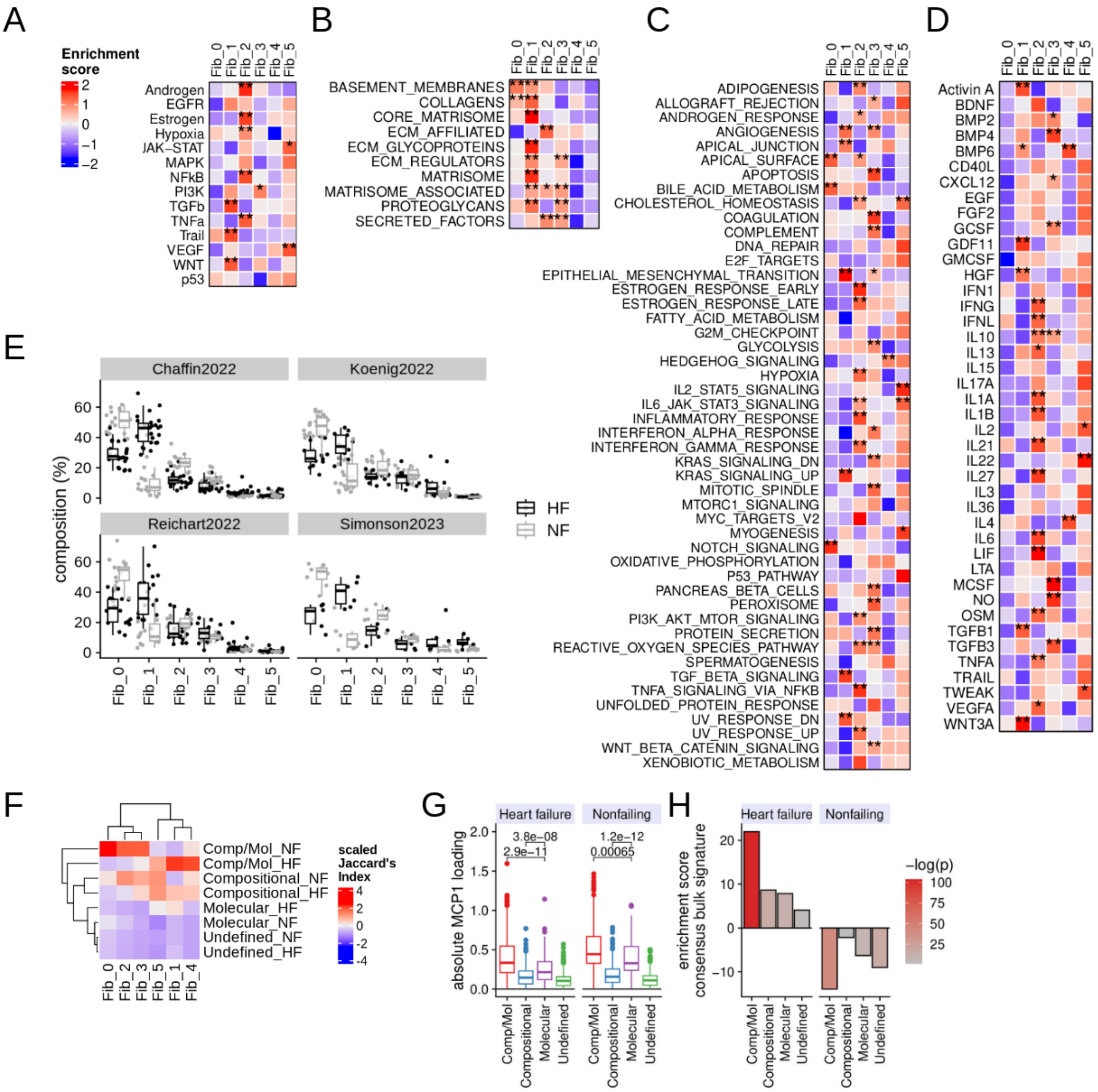

Supplementary Figure 11. Functional characterization of fibroblasts using single-nucleus RNAseq data. A-D) Different prior knowledge gene sets were enriched in pseudobulked cell state and patient profiles. Mean scaled enrichment scores across patients shown, t-test were performed on enrichment scores via one vs. all with adjusted p-value *<0.01, **0.001, ***0.0001. The different prior knowledge gene sets included (A) PROGENy pathways, (B) ECM genes (NABA geneset from MSIGDB), (C) MSIGDB Hallmarks, (D) Cytosig.

E) Sample composition of fibroblast cell states separated by HF (color) and studies (panels).

F) Jaccard Indices scaled per cell state to visualize intersections of top 150 state markers with division of labor groups.

G) Absolute loadings of multicellular program 1, comparing division of labor programs for heart failure and non-failing associated genes. Wilcoxon’s test p-values are provided for selected comparisons.

H) Enrichment scores (y-axis) for different division of labor programs associated with heart failure and non-failing genes in the bulk consensus signature of heart failure. Enrichment was performed using univariate linear models, with negative log10-transformed p-values displayed as coloring.

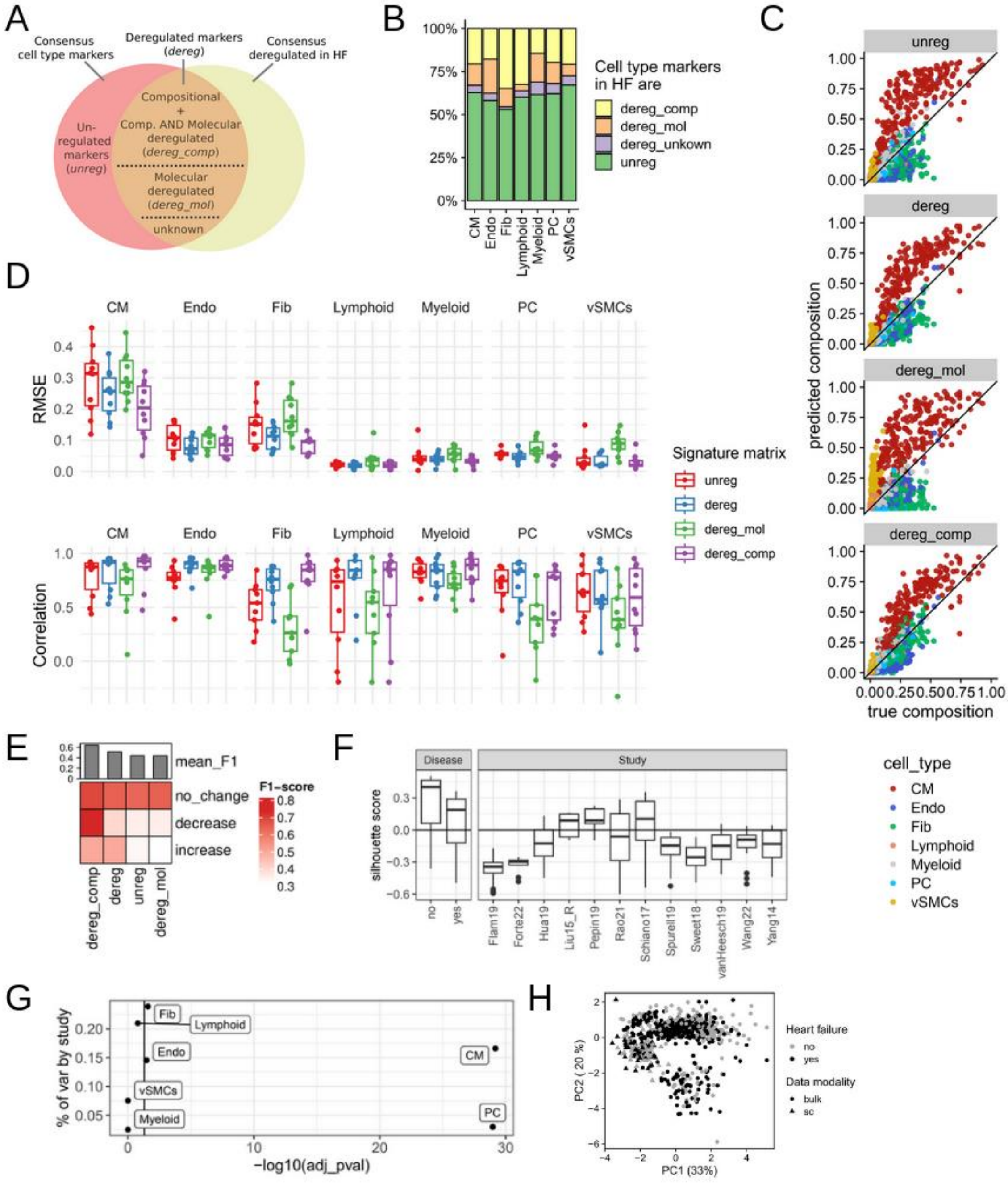

Supplementary Figure 12. Cell type deconvolution of pseudobulk and bulk.

A. Schematic of annotation of cell type markers deregulated in HF. Different subsets of markers and their deregulation characteristics were used to build signature matrices for cell type deconvolution. The abbreviations for the four resulting signature matrices are written in italic.

B. Quantification of annotations described in (A) per cell type.

C. Comparing true (x-axis) and estimated compositions (y-axis) from pseudobulked profiles of patients, colored by cell type. Each panel represents estimated compositions by using a different signature matrix.

D. Quantification of deconvolution performance per cell type and signature matrix. Root mean square error (RMSE, top) and Pearson's correlation (bottom) were calculated (each data point represents one study).

E. F1-scores for the prediction of cell type composition changes based on estimated compositions. Significant composition changes of the seven cell lineages were classified into three categories, *increase*, *decrease* or *no change* based on a t-test p-value <0.05. Top bar plot displays global average of F1 for each signature matrix.

F. Estimated cell type compositions from core HF bulk studies are compared for their clustering of HF status (left panel) and study label (right panel) via silhouette scores.

G. A linear mixed model was applied to meta-analyze composition changes of cell types in deconvoluted bulk studies. The variance explained by the study random effect (y-axis) is compared with the log-

transformed p-value of the cell type fixed effect (x-axis).

H. Principal component (PC) analysis of cell type composition profile of single-nucleus (sc) and bulk cohort. PC one and two (with explained variance) on x and y-axis, respectively.

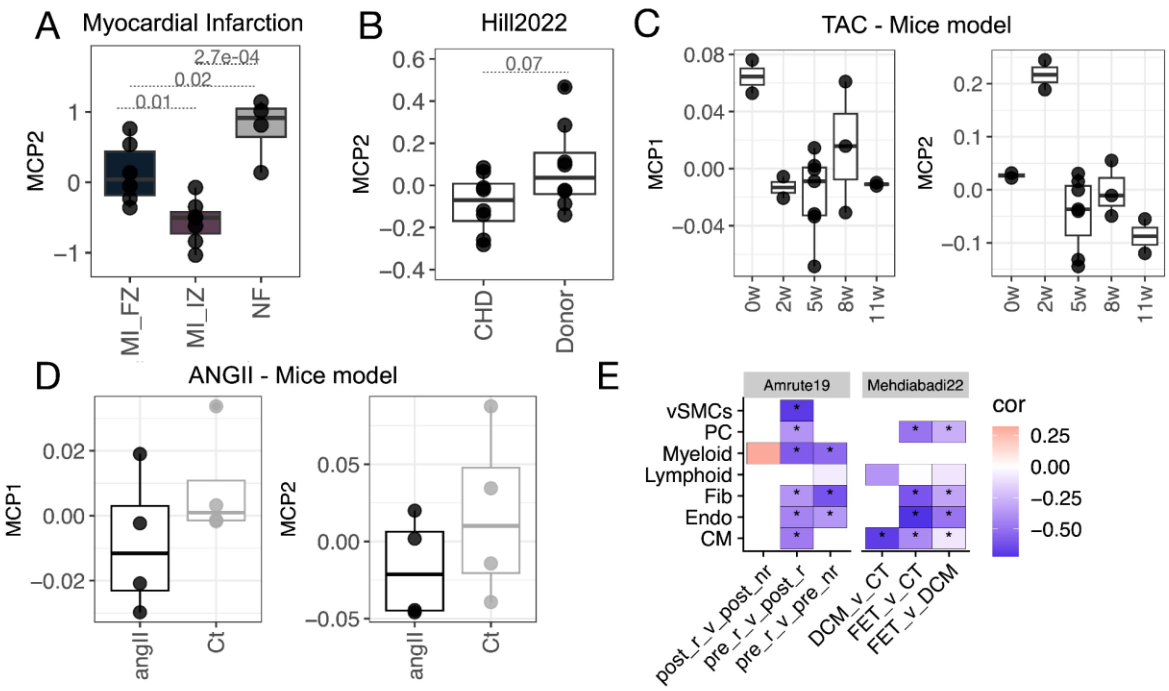

Supplementary Figure 13. Projection of tissue samples into the patient map of multicellular programs (MCPs) of HF.

A. MCP2 values of HF samples obtained from myocardial infarction (MI) patients at different time-points of the disease (FZ = fibrotic, IZ = ischemic) and control non-failing (NF) donor samples.

B. MCP2 values of HF samples obtained from congenital heart disease (CHD) and control non-failing donor patients.

C. MCP1 and 2 values of heart tissue samples obtained from mice with induced HF via transverse aortic constriction (TAC) at different time points.

D. MCP1 and 2 values of heart tissue samples obtained from mice with (Angiotensin II)-induced HF and control mice.

E. Correlating t-statistics of significantly deregulated genes reported by two individual studies per major cell lineage with gene loadings from the MCP1. Pearson correlation coefficients. * p-value <0.05. Since negative loadings are associated with HF, negative correlations indicate shared regulatory direction in HF.

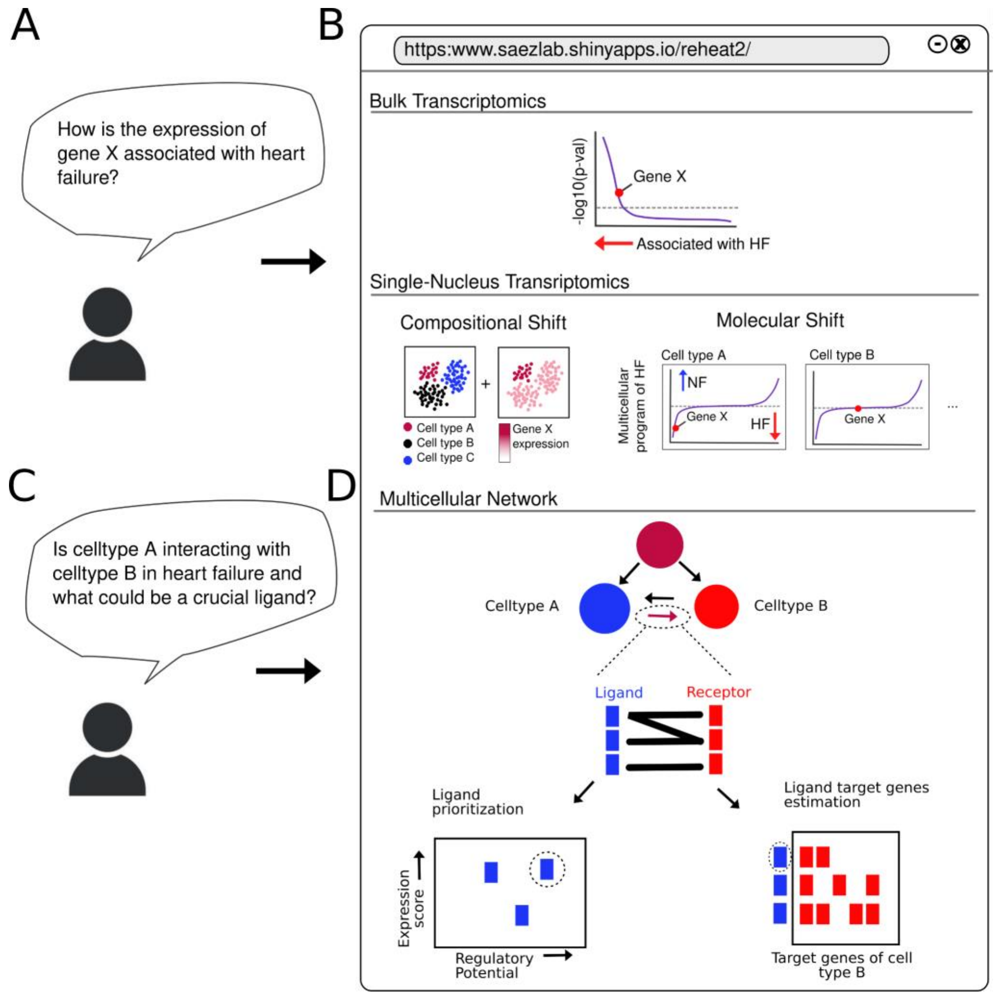

Supplementary Figure 14. User guide for ReHeaT2 web platform

A. Cardiovascular researchers can query genes of interest to explore their expression patterns in heart failure (HF).
B. ReHeaT2 (Reference of the HEArt failure Transcriptome) is web platform that provides a user-friendly query of single genes. Bulk transcriptomic analysis allows users to assess the statistical strength of a gene's association with HF. Study-specific log fold changes (HF vs. NF) illustrate variability across datasets, while a consensus HF signature ranks genes based on their combined p-value, providing an integrated measure of significance. Single-nucleus transcriptomics dissects whether a gene's deregulation is driven by changes in cell-type composition or by intrinsic molecular reprogramming, or both. Cell-type-specific expression patterns indicate whether a gene is a marker of a particular cell type, which, in combination with compositional shifts, suggests population-level regulation. Molecular regulation is further examined using MCP1 gene loadings, identifying cell-intrinsic transcriptional changes.
C. Users can analyze cell-cell interactions in HF by querying cell type pairs to explore their dependencies and potential mediating ligands.
D. The Multicellular Network tab highlights key edges in HF and non-failing heart networks. By selecting an edge of interest, users can generate ligand-receptor pair plots, ligand prioritization plots, and ligand-target gene visualizations.

2. Glossary

BH	Benjamini Hochberg
CM	Cardiomyocytes
DCM	Dilated cardiomyopathy
EC	Endothelial cell
HF	Heart failure
HCM	Hypertrophic cardiomyopathy
ICM	Ischemic cardiomyopathy
Fib	Fibroblast
MCP	Multicellular program
NF	Non-failing heart
NRVCM	Neonatal rat ventricular cardiomyocytes
Sn	Single-nucleus RNAseq
vSMC	Vascular smooth muscle cell

3. Supplementary references

1. Fleming, S. J. *et al.* Unsupervised removal of systematic background noise from droplet-based single-cell experiments using CellBender. *Nat. Methods* **20**, 1323–1335 (2023).
2. Schmidt, K. L. *et al.* The cell migration molecule UNC-53/NAV2 is linked to the ARP2/3 complex by ABI-1. *Development* **136**, 563–574 (2009).
3. Ninh, V. K. *et al.* Spatially clustered type I interferon responses at injury borderzones. *Nature* (2024) doi:10.1038/s41586-024-07806-1.
4. Tran, D. T., Batchu, S. N. & Advani, A. Interferons and interferon-related pathways in heart disease. *Front. Cardiovasc. Med.* **11**, 1357343 (2024).
5. Chen, Y.-M. *et al.* Effect of LYRM1 knockdown on proliferation, apoptosis, differentiation and mitochondrial function in the P19 cell model of cardiac differentiation in vitro. *J. Bioenerg. Biomembr.* **48**, 33–41 (2016).
6. Widden, H. & Placzek, W. J. The multiple mechanisms of MCL1 in the regulation of cell fate. *Commun. Biol.* **4**, 1029 (2021).
7. Esper, L. *et al.* Role of SOCS2 in modulating heart damage and function in a murine model of acute Chagas disease. *Am. J. Pathol.* **181**, 130–140 (2012).
8. Yuan, M. *et al.* Targeting SOCS2 alleviates myocardial fibrosis by reducing nuclear translocation of β-catenin. *Biochim. Biophys. Acta Mol. Cell Res.* **1871**, 119804 (2024).
