# Supplementary material for: A cross-study transcriptional patient map of heart failure defines conserved multicellular coordination in cardiac remodeling
Source: Nat Commun. 2025 Oct 31;16:9659. doi: 10.1038/s41467-025-62219-6 (PMC12579254; doi:10.1038/s41467-025-62219-6)
Supplement: Supplementary file 2 — Description of Additional Supplementary Files [file 41467_2025_62219_MOESM2_ESM.pdf]

Supplementary data file 1. Source links of used data

File name: supp_file_1_data_repositories.csv

Description: Source links for data repositories for core and supporting studies.

Supplementary data file 2. Consensus cell-type gene markers

File name: supp_file_2_consensus_markers.csv

Description: Results of the Fisher combined test aggregating cell-type markers across studies. Mean log-fold changes (LFC) and adjusted two-sided p-values are provided.

Supplementary data file 3. Differentially expressed genes between DCM and ICM or HCM patients.

File name: supp_file_3_etiologymodel.csv

Description: Gene level statistics from linear mixed models comparing etiologies using DCM tissue samples as references, including BH adjusted two-sided p-values.

Supplementary data file 4. Search terms for functional analysis

File name: supp_file_4_gene_set_query.csv

Description: Search terms Cardiomyocytes, Vasculature and Fibrosis gene sets to select genes sets from MSigDB for functional interpretation

Supplementary data file 5. Ligand-Receptor interaction in non-failing and failing tissue

File name: supp_file_5_LR_mcp1.csv

Description: Ligand-Receptor interactions between cell type pairs based on the assigned gene loadings within MCP1

Supplementary data file 6. Division of labor gene programs

File name: supp_file_6_dol_fib_genes.csv

Description: Results from the linear mixed model to assign division of labor groups to fibroblast genes within MCP1

Supplementary data file 7. Consensus signature of heart failure

File name: supp_file_7_cardiac_cells_signature_matrix_TPM.txt

Description: Genes ranked by consensus deregulation in HF, including MCP1 gene weights, log-fold changes for cell-type marker genes, and annotations of potential deregulation mechanisms.

Supplementary data file 8. Cardiac cell type signature matrix for deconvolution tasks

File name: supp_file_8_reheat2_stats_bulk.csv

Description: Cell type signature matrix of compositionally regulated genes with TPM normalized expression values from a healthy reference atlas

Supplementary data file 9. qPCR primers for in vitro experiments.

File name: supp_file_9_qpcr_primers.csv

Description: List of qPCR primers and their sequences used for evaluating target gene expression of ligand treated neonatal rat ventricular cardiomyocytes.
